# Inhibition of Allogeneic and Autologous T Cell Proliferation by Adipose-Derived Mesenchymal Stem Cells of Ankylosing Spondylitis Patients

**DOI:** 10.1155/2021/6637328

**Published:** 2021-03-12

**Authors:** Ewa Kuca-Warnawin, Magdalena Plebańczyk, Krzysztof Bonek, Ewa Kontny

**Affiliations:** ^1^Department of Pathophysiology and Immunology, National Institute of Geriatrics, Rheumatology and Rehabilitation, Warsaw 02-637, Poland; ^2^Department of Rheumatology, National Institute of Geriatrics, Rheumatology and Rehabilitation, Warsaw 02-637, Poland

## Abstract

**Background:**

In ankylosing spondylitis (AS), accompanied by chronic inflammation, T cell expansion plays a pathogenic role; the immunoregulatory properties of bone marrow-derived mesenchymal stem cells (BM-MSCs) are impaired, while functional characteristics of their adipose tissue-derived counterparts are (ASCs) unknown.

**Methods:**

We evaluated the antiproliferative activity of AS/ASCs, obtained from 20 patients, towards allogeneic and autologous T lymphocytes, using ASCs from healthy donors (HD/ASCs) as the reference cell lines. The PHA-activated peripheral blood mononuclear cells (PBMCs) were cocultured in cell-cell contact and transwell conditions with untreated or TNF + IFN*γ-* (TI-) licensed ASCs, then analyzed by flow cytometry to identify proliferating and nonproliferating CD4^+^ and CD8^+^ T cells. The concentrations of kynurenines, prostaglandin E_2_ (PGE_2_), and IL-10 were measured in culture supernatants.

**Results:**

In an allogeneic system, HD/ASCs and AS/ASCs similarly decreased the proliferation of CD4^+^ and CD8^+^ T cells and acted mainly via soluble factors. The concentrations of kynurenines and PGE_2_ inversely correlated with T cell proliferation, and selective inhibitors of these factors synthesis significantly restored T cell response. AS/ASCs exerted a similar antiproliferative impact also on autologous T cells.

**Conclusion:**

We report for the first time that despite chronic in vivo exposure to inflammatory conditions, AS/ASCs retain the normal capability to restrain expansion of allogeneic and autologous CD4^+^ and CD8^+^ T cells, act primarily via kynurenines and PGE_2_, and thus may have potential therapeutic value. Some distinctions between the antiproliferative effects of AS/ASCs and HD/ASCs suggest in vivo licensing of AS/ASCs.

## 1. Introduction

Ankylosing spondylitis (AS) is a rheumatic disease characterized by chronic inflammation and pathological new bone formation at axial joints with resulting spinal segment fusion. Peripheral arthritis, enthesitis, osteoporosis, and extraskeletal symptoms, such as involvement of the eye, skin, or gut, are also common [1]. It is proposed that immune barrier dysfunction (gut, skin) and/or aberrant immune reactions at sites of mechanical stress (entheses, vessel walls), together with mutual interactions between innate and adaptive immune mechanisms, are critical for triggering, development, and regulation of AS [2]. The striking association of AS with HLA-B∗27 alleles and epistatic interactions with alleles encoding endoplasmic reticulum aminopeptidases (ERAP) indicate a key pathogenic role of antigen presentation to T cells, followed by development and persistence of adaptive immune response, which involves primarily T cell subsets [3]. In AS patients, T cells predominate in early and active sacroiliitis and represent about 50% of cells infiltrating affected joints [4, 5]. Increased number and greater proliferation of CD4^+^ and CD8^+^ T cells were found in both the synovial fluid and peripheral blood of these patients [6–9]. Mesenchymal stromal/stem cells (MSCs), present in various tissues, are endowed with immunomodulatory potency. These cells are known to exert immunosuppressive effects on different immune cells, including T lymphocytes [10]. Acting via soluble mediators and cell contact-dependent pathways, MSCs inhibit activation, and proliferation of T cells suppress effector but protect or induce regulatory T cells (Treg) [11]. Among numerous soluble factors, prostaglandin E_2_ (PGE_2_) and indoleamine-2,3-dioxygenase (IDO)/kynurenine pathway are regarded as the main mediators of immunosuppressive activity of human MSCs, including inhibition of T cell proliferation [12–14]. To improve the therapeutic features of MSCs for successful clinical application, several strategies were developed, and preconditioning with proinflammatory cytokines, e.g., tumor necrosis factor (TNF) and interferon *γ* (IFN*γ*), is commonly used [15]. Importantly, bone marrow-derived MSCs (BM-MSCs) from AS patients show abnormalities in gene expression, secretory potential, and reduced immunomodulatory activity [16–18]. Moreover, BM-MSCs are suggested to contribute to abnormal bone homeostasis, characteristic of AS, because of their strong capacity to inhibit osteoclastogenesis as well as enhanced osteoblastogenic and adipogenic differentiation [19–21]. Therefore, in currently ongoing clinical trials allogeneic, but not autologous, MSCs of various tissue origin are used for AS patients' treatment. However, adipose-derived mesenchymal stem cells (ASCs), known to possess stronger immunosuppressive properties than BM-MSC [14], have not been tested yet [22]. Moreover, in rheumatic diseases, bone marrow is the site where inflammation takes place, which is in contrast to not affected peripheral adipose tissue [23]. Interestingly, promising results of the application of autologous stromal vascular fraction (SVF), containing adipose-derived mesenchymal stem cells (ASCs), suggest the therapeutic use of those cells in AS [24]. Unfortunately, the biology of ASCs of AS patients (AS/ASCs) is poorly understood, and data about immunomodulatory properties of AS/ASCs are missing. We have previously found that ASCs of AS patients (AS/ASCs) show certain abnormalities in the expression of surface markers and secretion of soluble factors but retain the capability to regulate the expression of T cell activation markers [25, 26]. Considering an important role of T cells in AS pathogenesis and the potential therapeutic application of autologous ASCs, in the present study, we have focused on further evaluation of the immunoregulatory potential of AS/ASCs, by assessing antiproliferative activity and mechanism (s) of action of these cells on allogeneic and autologous CD4^+^ and CD8^+^ T lymphocytes. To this aim, mitogen-stimulated peripheral blood mononuclear cells (PBMCs) were cocultured with untreated or TNF + IFN*γ* (TI) preconditioned ASCs, obtained from AS patients and healthy donors (HD); then, the proliferation of CD4^+^ and CD8^+^ T lymphocytes as well as the release of PGE2, kynurenines, and interleukin- (IL-) 10 was analyzed. The contribution of soluble mediators to immunomodulatory ASCs action was verified by preventing cell-cell contact, using the transwell system, and application of specific inhibitors of PGE_2_ and kynurenine synthesis.

## 2. Materials and Methods

### 2.1. Patients and Sample Collection

A group of 20 patients (8 females, 12 males) who fulfilled the ASAS (Assessment of SpondyloArthritis International Society) criteria for AS [27] were included in the study. Patients' characteristics are given in Table 1. This study meets all criteria contained in the Declaration of Helsinki and was approved by the Ethics Committee of the National Institute of Geriatrics, Rheumatology, and Rehabilitation, Warsaw, Poland (the approval protocol no: KBT-8/4/20016). All patients gave their written informed consent before enrolment.

### 2.2. ASC Isolation and Culture

Specimens of subcutaneous abdominal fat were taken from the patients by an 18 G needle biopsy. Tissue processing, ASC isolation, and culture were performed as described previously [28]. Five human adipose-derived mesenchymal cell lines (Lonza Group, Lonza Walkershille Inc., MD, USA; donor numbers: 0000440549, 0000410252, 0000535975, 0000605220, 0000550179) were used as a control. All experiments were performed using ASCs at 3–5 passages. ASCs were cultured in a complete culture medium composed of DMEM/F12 (PAN Biotech UK Ltd., Wimborn, UK), 10% fetal calf serum (FCS) (Biochrom, Berlin, Germany), 200 U/ml penicillin, 200 *μ*g/ml streptomycin (Polfa Tarchomin S.A., Warsaw, Poland), and 5 *μ*g/ml plasmocin (InvivoGen, San Diego, CA, USA). Preconditioning (“priming” or “licensing”) of ASCs with proinflammatory cytokines was performed by stimulation of the cells for 24 h with human recombinant tumor necrosis factor (TNF) and interferon *γ* (IFN*γ*) (both from R&D Systems, Minneapolis, MN, USA; each applied at 10 ng/ml). The concentration of TNF and IFN*γ* was determined based on previous publications [29–31].

### 2.3. Contacting and Noncontacting Cocultures of ASCs with Peripheral Blood Mononuclear Cells (PBMCs)

All cocultures were performed in the complete DMEM/F12 medium. ASCs (6 × 10^4^/well/2 ml of medium) were seeded into 24-well plates and stimulated with IFN*γ* and TNF (see above). PBMCs were isolated from buffy coats obtained from healthy male honorary blood donors (<60 years old) and AS patients, according to routinely applied procedure with the use of Ficoll-Paque (GE Healthcare, Uppsala, Sweden). After isolation, PBMCs (1.2 × 10^6^/well/2 ml of medium) were seeded either directly (contacting coculture) or on a 0.4 *μ*m pore size Transwell filters (MD24 with a carrier for inserts 0.4 MY, Thermo Fisher Scientific, Massachusetts, MA, USA) (noncontacting coculture) into 24-well plates with adherent ASCs (6 × 10^4^/well) and treated with 2.5 *μ*g/ml of phytohaemagglutinin (PHA, Sigma-Aldrich, St. Louis, MO, USA). After 5 days of coculture, culture supernatants (SNs) and PBMCs were harvested for further analysis, i.e., the measurement of the concentrations of soluble factors—kynurenines, PGE_2_, and IL-10 or flow cytometry, respectively. PBMCs cultured separately were used as control.

### 2.4. The Measurement of Soluble Factors in Culture Supernatants

Kynurenine concentration was measured spectrophotometrically as described elsewhere [32]. To this aim, SNs were mixed with 30% trichloroacetic acid at a 2 : 1 ratio and incubated for 30 min at 5°C, then centrifuged at 10000 × g for 5 min and finally diluted at a 1 : 1 ratio in Ehrlich's reagent (100 mg p-dimethyl benzaldehyde and 5 ml glacial acetic acid; Sigma-Aldrich, St. Louis, MO, USA). The optical density of the samples was measured at a wavelength of 490 nm. L-Kynurenine (Sigma-Aldrich, St. Louis, MO, USA) diluted in culture medium was used to prepare the standard curve. The concentrations of PGE_2_ and IL-10 were measured using commercially available kits—the Parameter kit (R&D Systems, Minneapolis, MN) and human IL-10 Enzyme-Linked Immunosorbent Assay (Thermo Fischer; cat. no. 88-7104-88), respectively. All measurements were done in duplicates.

### 2.5. Blocking Experiments

To investigate the role of PGE2 and kynurenines in the immunomodulatory capacity of ASCs, specific inhibitors of these factors synthesis, i.e., 10^−6^ M of indomethacin (Sigma-Aldrich, Germany) or 1 mM of 1-methyl-tryptophan (1-MT, Sigma-Aldrich, Germany), respectively, were added at the beginning of the cell culture periods. The above concentrations were selected from literature searches of previous studies [32–36]. After 48 hours of ASCs preincubation with specific inhibitors, PHA-activated and CFSE-stained PBMCs were added to the culture. Next, 1-MT or indomethacin was added again. The cultures were incubated for 5 days at 37°C in a humidified atmosphere of 5% CO_2_. At the end of the coculture, PBMCs were harvested for further cytometric analysis.

### 2.6. Flow Cytometry Analysis

#### 2.6.1. Identification of T Cell Subsets

PBMCs harvested from cultures were resuspended in 50 *μ*l of FACS buffer and stained for 30 min on ice for respective membrane antigens using fluorochrome-conjugated monoclonal antibodies specific for human: CD4–APC-Cy7 (BD Pharmingen, San Diego, CA, USA) or CD8-PerCP (eBioscience, San Diego, CA, USA). After the washing step, cells were acquired and analyzed using a FACSCanto cell cytometer and the Diva software. Appropriate isotype controls were used in all experiments.

#### 2.6.2. Proliferation Assay

For proliferation assay, PBMCs were stained with carboxyfluorescein diacetate succinimidyl ester (CFSE) (Thermo Fisher Scientific, Massachusetts, MA, USA), then stimulated with PHA and cocultured with ASCs as described above. Cells harvested from cultures were analyzed by flow cytometry to identify proliferating and nonproliferating cells. To characterize cellular proliferation response, the percentage of proliferating cells, proliferation index (PI), and replication index (RI) was calculated as described elsewhere [37], using the following mathematical formulas:

(1) $$\begin{array}{c} \text{PI}=\frac{\sum_{1}^{i}i\times \left(N_{i}/2^{i}\right)}{\sum_{1}^{i}\left(N_{i}/2^{i}\right)}. \end{array}$$

Proliferation index is for responding cells, an average number of a division they have undergone;*N*is the number of cells in the division, and*i*is the number of division.

(2) $$\begin{array}{c} \text{RI}=\frac{\sum_{1}^{i}N_{i}}{\sum_{1}^{i}\left(N_{i}/2^{i}\right)\;}. \end{array}$$

Replication index is for responding cells, fold expansion over the culture time;*N*is the number of cells in the division, and*i*is the number of division.

**Figure 1:**
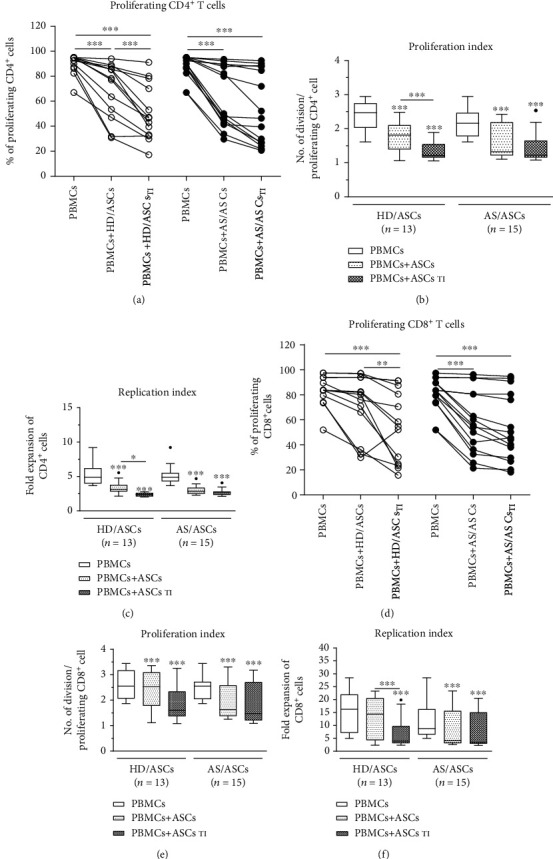
Inhibition of T cell proliferation by ASCs of healthy donors (HD) and AS patients. Peripheral blood mononuclear cells (PBMCs) obtained from 9 healthy donors (HD) were stimulated with PHA and cultured alone (control) or cocultured for 5 days with either untreated or TNF + IFN*γ*- (TI-) stimulated ASCs from 5 HD (HD/ASCs) or 10 AS patients (AS/ASCs). The proliferation of CD4^+^ (a–c) and CD8^+^ (d–f) T cells was analyzed by flow cytometry. Data are the results of the indicated number of experiments (*n*). (a, d) Lines between points identify cultures containing the same combination of ASCs and PBMCs. (b–f) Results are expressed as the median (horizontal line) with interquartile range (IQR, box), lower and upper whiskers (data within 3/2×IQR) and outliers (points) (Tukey's box). ^∗^*P* = 0.05–0.01, ^∗∗^*P* = 0.01‐0.001, and ^∗∗∗^*P* = 0.001‐0.0001 for intragroup comparisons (cell cocultures vs. control separate cultures and as indicated). The intergroup (HD vs. AS) differences were statistically insignificant.

Exemplary histograms showing the proliferation of PHA-stimulated PBMCs in various culture variants are shown in Figure 1S (supplementary materials).

### 2.7. Data Analyses

Data were analyzed using the GraphPad Prism software version 7. The Shapiro-Wilk test was used as a normality test. One-way analysis of variance (ANOVA) with repeated measures and post hoc Tukey test was used to assess the effect of untreated and TNF/IFN*γ*-treated ASCs on target cells, to compare contacting versus (vs.) noncontacting cocultures and allogeneic vs. autologous cocultures. The Mann–Whitney test was applied to analyze differences between HD/ASCs and AS/ASCs.

Parametric (Pearson's linear) and nonparametric (Spearman's rank) correlation tests were used to assess an association between analyzed parameters. Probability values less than 0.05 were considered significant.

## 3. Results

### 3.1. Patients

The patient cohort was heterogeneous concerning demographic and clinical data (Table 1). Ninety percent of patients were HLA-B27 positive, 40% of them had ocular symptoms (uveitis), and 10% had peripheral arthritis. They were mostly treated with nonsteroid anti-inflammatory drugs (NSAIDs), while application of nonbiologic disease-modifying antirheumatic drugs (DMARDs) and glucocorticosteroids was less frequent.

### 3.2. Inhibition of T Cell Proliferation by ASCs

In control, separately cultured, PHA-treated PBMCs (PBMCs_PHA_), the majority of CD4^+^ and CD8^+^ T cells proliferated (mean ± SEM = 88.7 ± 1.6% and 81.9 ± 2.4%, respectively), but considerable PBMC donor-dependent variation was observed (Figures 1(a) and 1(d)). As showed in Figure 2S in supplementary materials, also the degree of inhibition of proliferative response was determined by the individual specificity of the donors of PBMCs, because the same HD/ASCs or AS/ASC lines exerted nonidentical effects on PBMCs originated from different donors (compare donors no. 1 and no.2). Therefore, HD/ASC and AS/ASC activities were evaluated in the same experiments, using PBMCs from the same donors. In the presence of both untreated and TI-stimulated ASCs, the number of proliferating T cells of both subsets and the number of division per proliferating cell (PI) as well as fold expansion of these cells (RI) decreased significantly. Importantly, HD/ASCs and AS/ASCs exerted similar inhibitory effects (Figures 1(a)–1(f)). In the cocultures with HD/ASCs and HD/ASCs_TI_ vs. AS/ASCs and AS/ASCs_TI_, the number of proliferating CD4^+^ cells was inhibited by 23.1 ± 5.8% and 44.2 ± 6.2% vs. 29.3 ± 5.8% and 40.8 ± 7.4%, respectively, while the number of proliferating CD8^+^ cells was reduced by 14.3 ± 4.6% and 40.3 ± 7.7% vs. 29.4 ± 5.7% and 34.9 ± 6.2%, respectively (mean ± SEM; data not shown). There were no significant differences between the effects exerted by untreated and TI-treated AS/ASCs (Figures 1(a)–1(f)), while TI-treated HD/ASCs were more potent inhibitors of T cell proliferation than untreated HD/ASCs (Figures 1(a)–1(d) and 1(f)).

### 3.3. Contribution of Cell-to-Cell Contact and Soluble Factors to the Antiproliferative Effect of ASCs

**Figure 2:**
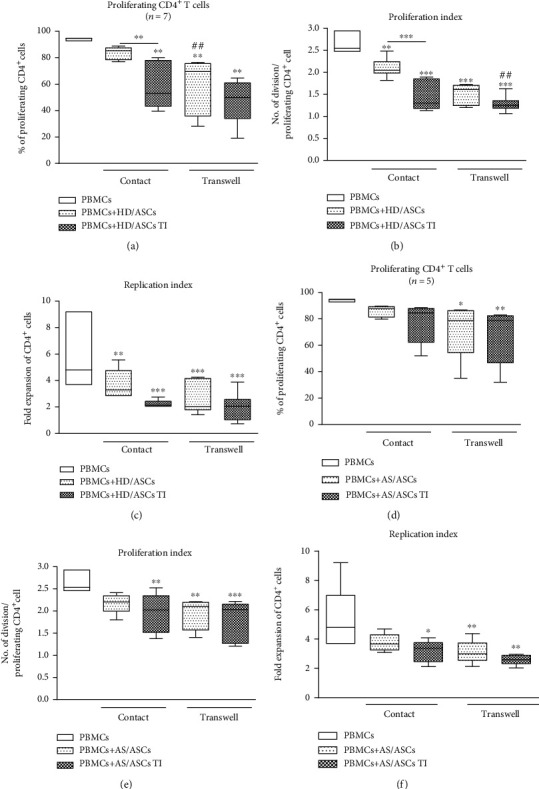
Comparison of ASC inhibitory effects on CD4^+^ T cell proliferation in contact and transwell cocultures. PBMCs, isolated from 3 healthy blood donors and activated with PHA, were cultured separately (control) or cocultured with ASCs from 4 healthy donors (HD/ASCs) or 5 AS patients (AS/ASCs) in conditions allowing direct cell contact or in a transwell system. Untreated and TNF + IFN*γ-* (TI-) treated ASCs were used. The proliferation of CD4^+^ T cells was analyzed by flow cytometry. Data are the results of the indicated number of experiments (*n*) and are shown as the Tukey's boxes. ^∗^*P* = 0.05‐0.01, ^∗∗^*P* = 0.01‐0.001, and ^∗∗∗^*P* = 0.001‐0.0001 for comparison of cell cocultures vs. control separate cultures and as indicated. ^##^*P* = 0.01‐0.001 for comparison of contact vs. transwell cocultures.

**Figure 3:**
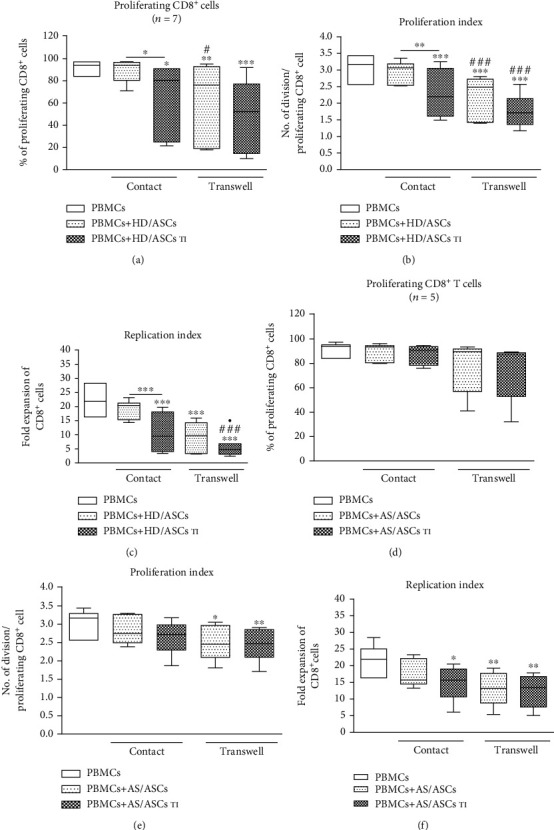
Comparison of ASC inhibitory effects on CD8^+^ T cell proliferation in contact and transwell cocultures. Cell origin and culture conditions as in Figure 2, except that CD8+ T cells were analyzed. ^∗^*P* = 0.05‐0.01, ^∗∗^*P* = 0.01‐0.001, and ^∗∗∗^*P* = 0.001‐0.0001 for comparison of cell cocultures vs. control separate cultures and as indicated. ^#^*P* = 0.05‐0.01 and ^###^*P* = 0.001‐0.0001 for comparison of contact vs. transwell cultures.

In the coculture conditions allowing or preventing (transwell) direct cell-to-cell contact, untreated and TI-treated AS/ASCs similarly reduced proliferation of CD4^+^ (Figures 2(d)–2(f)) and CD8^+^ (Figures 3(d)–3(f)) T cells, while the inhibitory effect of HD/ASCs was even more potent in transwell than cell-to-cell contact cultures, and such differences were observed in the case of CD4^+^ (Figures 2(a)–2(c)) and CD8^+^ (Figures 3(a)–3(c)) T cells. These results point to secretory factors as the critical mediators of ASCs triggered antiproliferative effects.

### 3.4. Upregulation of Kynurenines and PGE_2_ in the Cocultures of ASCs with PBMCs

**Figure 4:**
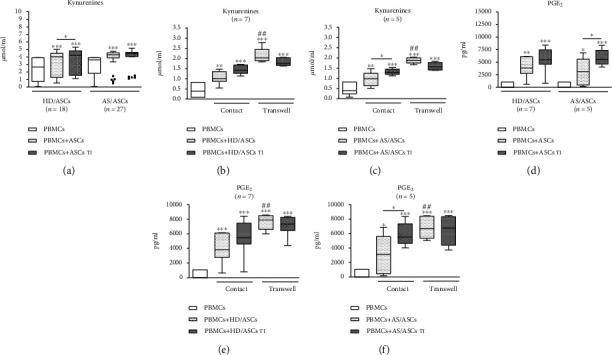
Upregulation of kynurenines and prostaglandin E_2_ (PGE_2_) synthesis in the contacting and noncontacting cocultures of PBMCs with ASCs. Cells were prepared and cocultured as described in Figures 1(a) and 1(d) and Figures 2(b), 2(c), 2(e), and 2(f). PBMCs were isolated from peripheral blood of 11 (a) or 5-7 (b–e) healthy blood donors. Five HD/ASC lines and AS/ASCs obtained from 12 (a) or 5-7 (b–e) patients were used. The concentrations of kynurenines and PGE_2_ in culture supernatants were measured as described in Material and Methods. Data are shown as the Tukey's boxes; the number of performed experiments (*n*) is shown. ^∗^*P* = 0.05‐0.01, ^∗∗^*P* = 0.01‐0.001, and ^∗∗∗^*P* = 0.001‐0.0001 for comparison of cell cocultures vs. control separate cultures and as indicated. ^##^*P* = 0.01‐0.001 and ^###^*P* = 0.001‐0.0001 for comparison of contact vs. transwell cultures.

Untreated PBMCs produced little to moderate quantity of kynurenines which upon activation rose significantly (mean ± SEM, 1.69 ± 0.25 vs. 2.56 ± 0.32 *μ*mol/ml, *P* = 0.0011 for untreated PBMCs vs. PBMCs_PHA_). A similar, significant increase of PGE_2_ level was found as well (no secretion vs. 456.2 ± 163 pg/ml, *P* = 0.016 for untreated PBMCs vs. PBMCs_PHA_). According to our previous findings, both HD/ASCs and AS/ASCs secrete kynurenines (2.5 ± 0.3 vs. 0.4 ± 0.2 *μ*mol/ml, *P* < 0.0001) and PGE_2_ (134 ± 27 vs. 357 ± 120 pg/ml, *P* = 0.35) spontaneously, and the release of these mediators increases upon TI pretreatment to the levels lower to similar to that found in present coculture experiments (kynurenines 4.3 ± 0.4 vs. 0.8 ± 0.3 *μ*mol/ml, *P* < 0.0001; PGE_2_315 ± 61 vs. 811 ± 246 pg/ml, *P* = 0.23) [24]. However, these indicative data can not be referred directly to present coculture conditions, because ASCs originated from other donors. Compared with separately cultured PBMCs_PHA_, in the cocultures of these cells with both untreated and TI-stimulated HD/ASCs and AS/ASCs, significant increases of kynurenines and PGE_2_ production were observed (Figures 4(a) and 4(d)). In comparison to untreated ASCs, the addition of TI-treated HD/ASCs triggered the significantly higher generation of kynurenines (a similar tendency was noticed for PGE_2_ secretion), while in the presence of TI-treated AS/ASCs only higher release of PGE_2_ was found. However, no significant differences between the magnitude of kynurenines and PGE_2_ elevation in the presence of HD/ASCs versus AS/ASCs were found. By contrast, in the same culture conditions secretion of IL-10 did not rise (Figure 3S).

### 3.5. Contribution of Cell-to-Cell Contact and Soluble Factors to Upregulation of Kynurenines and PGE_2_ by ASCs

The elevation of kynurenines and PGE_2_ production by untreated HD/ASCs and AS/ASCs was significantly higher in transwell than cell-to-cell contact cocultures with PBMCs_PHA_, and a similar tendency was observed in cocultures containing TI-treated ASCs (Figures 4(b), 4(c), 4(e), and 4(f), respectively), suggesting an important role of soluble factors in upregulation of both kynurenines and PGE_2_.

### 3.6. Association of the Secretion of Soluble Factors with the Antiproliferative Effect of ASCs

**Figure 5:**
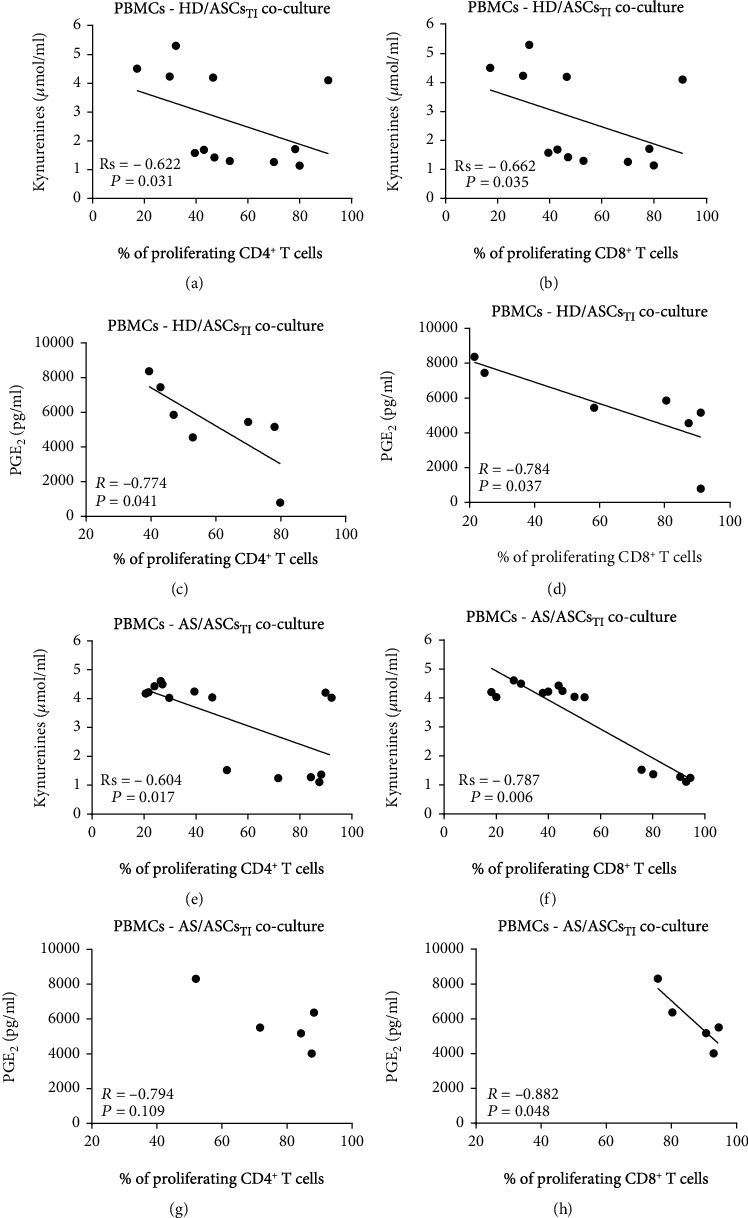
Correlation of kynurenines and PGE_2_ concentrations with the number of proliferating T cells. PBMCs stimulated with PHA were cocultured with TNF + IFN*γ-* (TI-) treated ASCs from HD (a–d) and AS patients (e–h). The proliferation of CD4^+^ (a, c, e, g) and CD8^+^ (b, d, f, h) cells was evaluated, and the measurement of kynurenines and PGE_2_ concentrations was performed as described in Materials and Methods. Spearman's rank (Rs) (a, b, e, f) and Pearson's (*R*) (c, d, g, h) correlation coefficients and *P* values are shown. Other explanations as in Figure 1.

**Figure 6:**
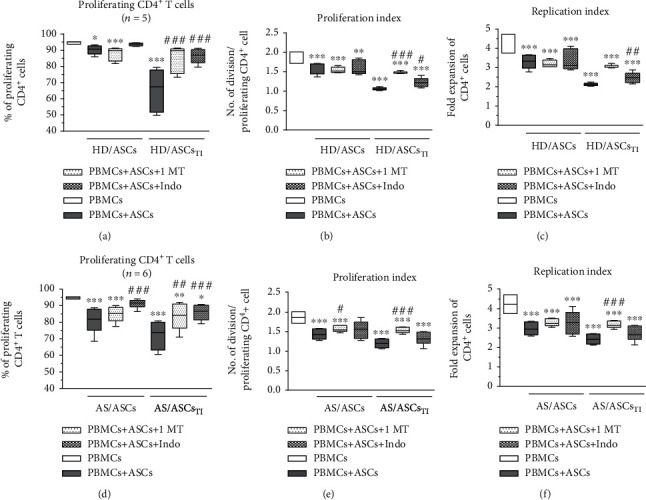
Selective inhibitors of kynurenines (1-MT) and PGE_2_ (indomethacin) synthesis counteract inhibitory effect of ASCs on proliferation of CD4^+^ T cells. Cell preparation and culture conditions as in Figure 1. The concentrations and time of cell treatment with 1-MT and indomethacin (Indo) are described in Materials and Methods. Data are shown as the Tukey's boxes; the number of performed experiments (*n*) is shown. ^∗^*P* = 0.05‐0.01, ^∗∗^*P* = 0.01‐0.001, and ^∗∗∗^*P* = 0.001‐0.0001 for comparison of cell cocultures vs. control separate cultures. ^#^*P* = 0.05‐0.01, ^##^*P* = 0.01‐0.001, and ^###^*P* = 0.001‐0.0001 for comparison of inhibitor treated vs. untreated cocultures. Other explanations as in Figure 1.

**Figure 7:**
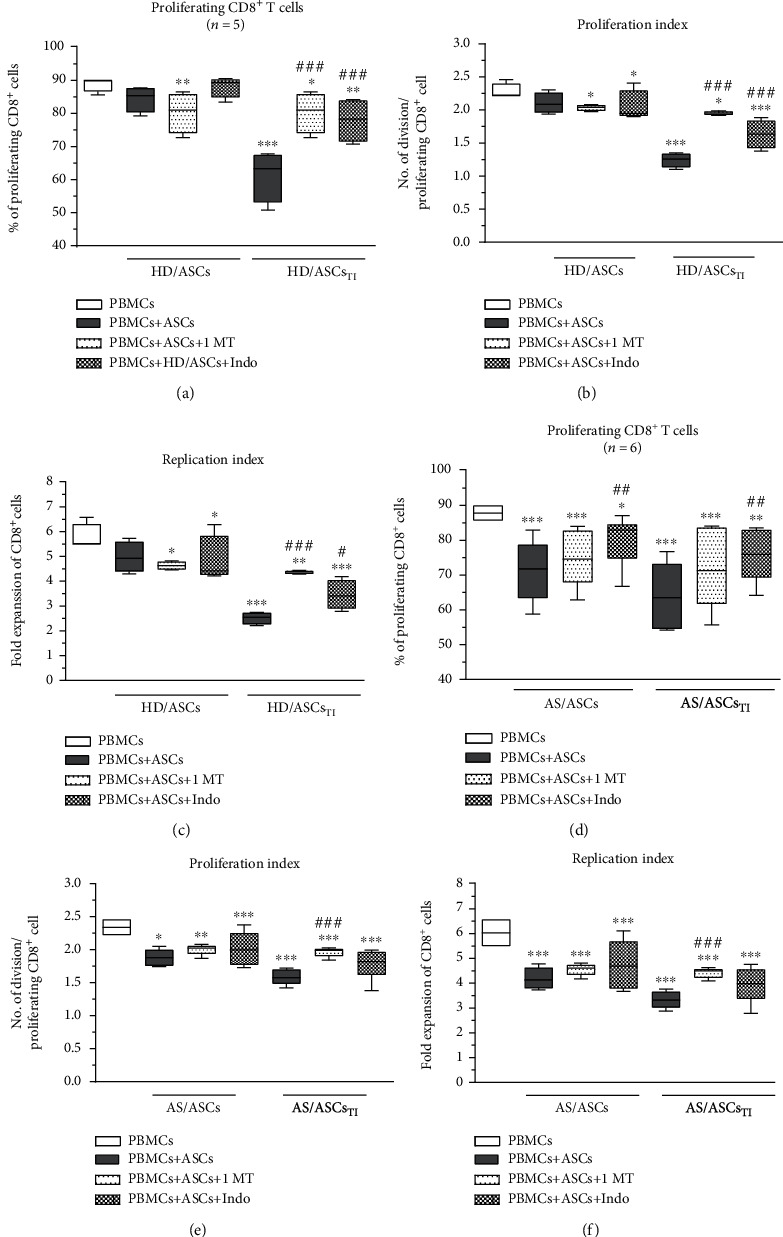
Selective inhibitors of kynurenines (1-MT) and PGE_2_ (indomethacin) synthesis counteract the inhibitory effect of ASCs on the proliferation of CD8^+^ T cells. Cell preparation, culture conditions, concentrations, and time of cell treatment with 1-MT and indomethacin (Indo) and other explanations as in Figure 6, except that CD8+ T cells were analyzed.

As shown in Figure 5, in the cocultures of PBMCs_PHA_ with TI-treated HD/ASCs and AS/ASCs, the concentration of kynurenines inversely correlated with the percentage of proliferating CD4^+^ (a, e) and CD8^+^ (b, f) T cells. A similar relationship was observed for untreated AS/ASCs (Figure (Figure 4S, panels E, F). However, in the case of HD/ASCs, it was statistically significant only for CD8^+^, but not CD4^+^, cell proliferation (Figure 4S, panels B and A, respectively). A negative correlation between the percentage of proliferating CD4^+^ and CD8^+^ T cells and PGE_2_ secretion was also found, but due to a small number of experiments, it does not always reached its statistical significance. Nevertheless, it was noticed only in the presence of TI-treated (Figures 5(c), 5(d), and 5(h); the similar trend in (g)) but not untreated (Figure 4S, panels C, D, G, H) ASCs. By contrast, secretion of IL-10 failed to show an inverse correlation with the number of proliferating T cells (Figure 5S), and in the cocultures of PBMCs_PHA_ with HD/ASCs_TI_ secretion of IL-10 correlated even positively with the number of proliferating CD4^+^ T cells (Figure 5S, panel C). These results point out kynurenines and PGE_2_, but not IL-10, as likely mediators of the antiproliferative capacity of ASCs. To verify this, we performed blocking experiments in which selective inhibitors of kynurenines (1-MT) and PGE_2_ (indomethacin) generation were used (Figures 6 and 7).

Both 1-MT and indomethacin partly, but significantly, abolished the antiproliferative effect of TI-treated HD/ASCs on CD4^+^ and CD8^+^ cells, increasing the number of proliferating cells as well as proliferation and replication indices. By contrast, applied inhibitors failed to reverse the weak inhibitory effect exerted by untreated HD/ASCs on the proliferation of these cells (Figures 6(a)–6(c) and Figures 7(a)–7(c), respectively). As for the antiproliferative capacity of AS/ASCs, 1-MT counteracted the effects of TI-treated cells on CD4^+^ (Figures 6(d)–6(f)) and CD8^+^ (Figures 7(d)–7(f)) T lymphocytes by increasing significantly the number of proliferating T cells and/or proliferation and replication indices, respectively. Indomethacin, in turn, almost completely reverted the number of proliferating CD4^+^ and CD8^+^ cells which was significantly reduced in the presence of both TI-treated and untreated AS/ASCs.

### 3.7. Immunoregulatory Effects of AS/ASCs toward Autologous PBMCs

**Figure 8:**
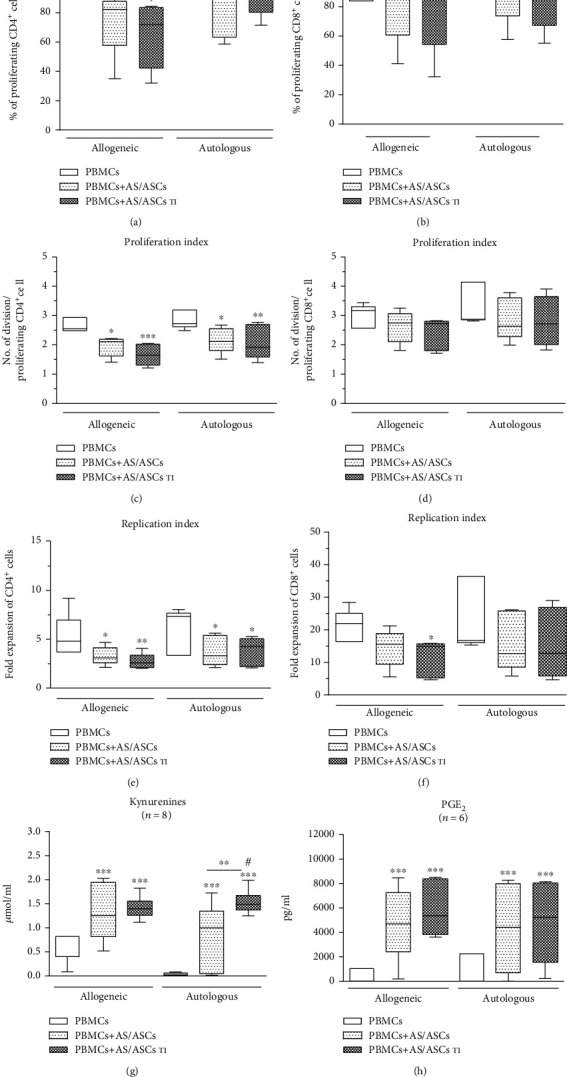
Comparison of immunomodulatory effects of AS/ASCs cocultured with allogeneic and autologous PBMCs. Cells were prepared and cocultured as described in Figure 1. Both allogeneic and autologous PHA-activated PBMCs were used as the target cells. Data are shown as the Tukey's boxes; the number of performed experiments (*n*) is shown. ^∗^*P* = 0.05‐0.01, ^∗∗^*P* = 0.01‐0.001, and ^∗∗∗^*P* = 0.001‐0.0001 for comparison of cell cocultures vs. control separate cultures and as indicated. ^#^*P* = 0.05‐0.01 for comparison of allogeneic vs. autologous cocultures.

Comparison of simultaneously performed cocultures of untreated and TI-treated AS/ASCs with allogeneic or autologous PBMCs_PHA_ demonstrated that in both systems AS/ASCs exerted similar antiproliferative effects on CD4^+^ and CD8^+^ T cells (Figures 8(a)–8(f)), as well as elevated the concentrations of kynurenines and PGE_2_ to similar levels (Figures 8(g) and 8(h), respectively). Besides, TI-treated AS/ASCs triggered an even significantly higher increase of kynurenines in the cocultures of autologous than allogeneic PBMCs (Figure 8(g)).

## 4. Discussion

It is well established that MSCs play a significant immunoregulatory role by damping down the activation of various immune cells, including T lymphocytes, monocytes/macrophages, and dendritic cells. Among numerous regulatory activities, immunosuppressive effects of MSCs on T cell proliferation have been confirmed both in vitro and in vivo conditions [10–14, 32, 38]. In ankylosing spondylitis, T cell expansion, noticed locally and in the periphery, is thought to play a pathogenic role [4–9]. Importantly, BM-MSCs of AS patients have some functional defects and are thought to contribute to pathological bone homeostasis [16–21]. It is worth noting that bone marrow is more prone to chronic inflammation than adipose tissue. All these observations may indicate a diminished therapeutic utility of autologous BM-MSCs in AS. Numerous data demonstrate that the immunomodulatory efficacy of MSCs from different tissue sources is not equal, and adipose tissue-derived MSCs (ASCs) have stronger immunosuppressive properties than BM-MSCs [14, 39]. Unfortunately, ASCs of AS patients have not been investigated in this regard. Our previous findings suggested that at least some AS/ASC properties may be changed, because of the reduced basal level of some surface molecules, i.e., CD90 and intracellular adhesion molecule 1 (ICAM-1) on AS/ASCs, impaired basal secretion of kynurenines and galectin-3 by these cells, but the normal capability to regulate expression of activation markers on allogeneic T cells was revealed [25, 26]. Therefore, in the present study, we have further evaluated the functionality of AS/ASCs by investigating their capability to control the proliferation of T cells, using HD/ASCs as the reference cell lines. Importantly, we found that in the allogeneic system both HD/ASCs and AS/ASCs inhibited the number of proliferating CD4^+^ and CD8^+^ T cells with similar potency (Figures 1(a) and 1(d)). Besides, a significant decrease in the number of divisions per proliferating CD4^+^ and CD8^+^ cell was also observed (Figures 1(b) and 1(e)), and consequently, both events resulted in the reduction of T cell expansion (Figures 1(c) and 1(f)). Our results are consistent with the observations of others about MSC properties in general [10–14, 40, 41], but demonstrating AS/ASCs as the effective suppressors of the proliferation of allogeneic CD4^+^ and CD8^+^ T cells is a new finding.

To provide successful immunomodulation, MSCs employ direct cell-to-cell interactions and soluble factors [10, 11, 14, 42, 43]. Similar to other reports [34, 44], we observed that the antiproliferative effect of HD/ASCs and AS/ASCs on both CD4^+^ and CD8^+^ T cells was mediated mostly by soluble factors (Figures 2 and 3). Further experiments identified kynurenines and PGE_2_ as critical contributors to the antiproliferative action of ASCs of both healthy donors and AS patients. First, we found that the concentrations of kynurenines and PGE_2_ (Figures 4(a) and 4(d)), but not anti-inflammatory cytokine—IL-10 (Figure 3S), rose significantly in the cocultures of PBMCs_PHA_ with untreated as well as TI-stimulated HD/ASCs and AS/ASCs. Secondly, in the presence of TI preconditioned HD/ASCs and AS/ASCs, the concentrations of both factors, especially kynurenines, inversely correlated with the number of proliferating CD4^+^ and CD8^+^ cells (Figure 5), while no such association was found in the case of IL-10 (Figure 5S). Finally, the antiproliferative impact of HD/ASCs and AS/ASCs was partly, but significantly, abolished by selective inhibition of kynurenine and PGE_2_ generation (Figures 6 and 7). Our observations are consistent with other reports proving the most critical role of the kynurenine pathway for ASC inhibition of T cell proliferation in humans [32, 44, 45]. In this pathway, indoleamine 2,3-dioxygenase (IDO) catabolizes the essential amino acid tryptophan (Trp), resulting in local Trp deprivation and kynurenine generation that eventually lead to suppression of T cell proliferation, these cell apoptosis and formation of Treg cells [46, 47]. The kynurenine pathway can be blocked by a Trp analog, 1-methyl-tryptophan (1-MT) which binds to the ferrous IDO complex but, due to the additional methyl group, cannot be catalytically converted to kynurenines [48]. Similar to the kynurenine pathway, also PGE2 was reported to mediate the antiproliferative effects of various types of MSCs on T cells [49–51]. Prostaglandin E_2_, one of the most potent members of the prostaglandin family, is known to regulate a multitude of events in T cell activation, including cell cycle arrest [52]. Like other prostanoids, PGE_2_ is synthesized from arachidonic acid on a metabolic pathway dependent on the enzymatic activity of cyclooxygenase (COX) isoenzymes, mainly COX-1 and COX-2 [53]. Indomethacin, a nonsteroidal anti-inflammatory drug, is a nonselective inhibitor of COX-1 and COX-2 activity and, consequently, an inhibitor of prostaglandin generation [51]. Interestingly, PGE_2_ was reported to induce IDO/kynurenine pathway in tolerogenic dendritic cells and to synergize with this pathway in the immunosuppression of NK cells by MSCs [36, 54]. Thus, both pathways may cooperate in mediating the antiproliferative effects of ASCs on T cells as well. Based on the present results of blocking experiments and the correlation analysis and in line with other reports concerning various types of MSCs [44, 45, 49, 50], we point out kynurenines and PGE_2_ as the important mediators of antiproliferative action of not only HD/ASCs but also AS/ASCs.

It should be clarified that because both PBMCs_PHA_ and ASCs generated kynurenines and PGE_2_, in our experimental setting, both 1-MT and indomethacin were present during the whole period of cell coculture. As mentioned in Results (Section 3.4), the basal release of kynurenines and PGE_2_ by resting PBMCs was low, significantly elevated upon PHA-treatment, and further augmented in the presence of untreated and TI-treated HD/ASCs and AS/ASCs (Figures 4(a) and 4(d)). On the other hand, we have previously reported that, compared to HD/ASCs, the spontaneous generation of kynurenines by AS/ASCs is impaired, while the basal synthesis of PGE_2_ is normal. Also, we have noticed that these differences between AS/ASCs and HD/ASCs persist even upon TI stimulation which significantly elevates the generation of both kynurenines and PGE_2_ [26]. However, despite basal differences observed in separate cultures, present results show that in cocultures with target cells AS/ASCs are at least as productive as HD/ASCs (Figures 4(a) and 4(d)).

As mentioned before, the execution of MSCs immunomodulatory potential is a complex process involving various soluble mediators as well as direct MSCs interaction with immune cells. In contrast to the transwell system, in the cell contacting cocultures, the degree of T cell proliferation inhibition and the levels of immunosuppressive mediators result from complex and multidirectional interactions between MSCs and different types of cells in the PBMCs pool. Present results showed (Figures 2 and 3) that the inhibitory effect of HD/ASCs on T cell proliferation was weaker in cell contacting than transwell cocultures, implying that interactions of HD/ASCs with other cell types may counteract, and thus supervise, the antiproliferative effect of these cells. A similar tendency was observed in the case of AS/ASCs, but the differences were statistically insignificant, suggesting weaker control of these cells' activity. Providing prosurvival signals may likely contribute to this event. For example, it was reported that in PBMCs-ASCs, coculture T, B, and NK cells adhere to ASCs, and part of bound T cells is kept in an activated, proliferative state. Interestingly, these cells express a high level of endoglin which switches transforming growth factor *β* (TGF*β*) signaling from anti- to proproliferative pathway [55]. Moreover, BM-MSCs were found to support B cell viability through direct cell contact, leading to upregulation of vascular endothelial growth factor (VEGF) which protects B cells against apoptosis [56]. In the cocultures of mononuclear cells with MSCs, even the density of MSC monolayer may generate prosurvival signals, because the decrease of monolayer density was followed by the dose-dependent increase in lymphocyte proliferation [57]. Finally, the low expression level of various surface molecules that mediate cell-to-cell contact and contribute to the immunosuppressive properties of MSCs [58] may also affect the antiproliferative activity of MSCs, which was proven in the case of CD90 [59]. Although we found that, in general, HD/ASCs and AS/ASCs exerted comparable inhibitory effects on T cell proliferation and acted mostly via soluble factors, kynurenines and PGE_2_, some dissimilarities between these cell lines were also observed. First, TI-priming significantly enhanced the antiproliferative impact of HD/ASCs, but not AS/ASCs, on both CD4^+^ and CD8^+^ cells (Figure 1). Similarly, an inverse correlation between the concentration of kynurenines and the number of proliferating CD4^+^ and CD8^+^ cells was more evident in cocultures containing TI-licensed (Figures 5(a) and 5(b)) than untreated HD/ASCs (Figure 3S, panels A, B). By contrast, in cocultures comprising untreated and TI-treated AS/ASCs, these associations were comparable (Figures 5(e) and 5(f) versus Figure 4S, panels E, F). Thus, AS/ASCs seem to be less sensitive than HD/ASCs to TI-triggered enhancement of their functions. The priming of MSCs with proinflammatory cytokines, a procedure named “licensing,” is one of several strategies to generate highly functional MSCs for therapeutic application [39]. Licensing of ASCs with TNF and/or IFN*γ* was reported to enhance the immunosuppressive functions of these cells, including inhibition of T cell proliferation [60, 61], which is in line with present results concerning HD/ASCs. This procedure is believed to mimic the tissue inflammatory milieu, known to promote the immunomodulatory function of MSCs [39]. Because ASCs of AS patients have been exposed in vivo to a chronic inflammatory microenvironment, and in vitro are less sensitive than ASCs of healthy donors to TI-licensing, they are presumably in vivo licensed cells of well-developed antiproliferative abilities.

Intriguingly, the correlation analysis and results of blocking experiments suggest that the contribution of kynurenines and PGE_2_ to the antiproliferative activity of ASCs of healthy donors and AS patients is not equal. Although both kynurenines and PGE_2_ were found to be essential mediators of the activity of TI-primed HD/ASCs (Figures 5(a)–5(d), 6(a)–6(c), and 7(a)–7(c)), they play smaller, if any, role in mediating weaker antiproliferative action of untreated HD/ASCs (Figures 6(a)–6(c), 7(a)–7(c), and 3S, panels A-D). Regarding AS/ASCs, kynurenines turned out to be the mediators of the antiproliferative activity of both TI-treated and, to a lesser extent, also untreated cells, influencing all tested parameters of proliferative response (Figures 5(e)–5(h), 6(d)–6(f), 7(d)–7(f), and 3S, panels E, F), while the contribution PGE_2_ to these cells function was restricted, since indomethacin counteracted the inhibitory effect of untreated and TI-treated AS/ASCs on the number of proliferating T cells only (Figures 6(d)–6(f) and 7(d)–7(f)).

Because it has previously been reported that in vitro preactivated T cells are partly and in a time-dependent manner, resistant to antiproliferative action of ASCs [62], we finally check whether AS/ASCs can inhibit proliferation of autologous T lymphocytes—the cells chronically exposed in vivo to the inflammatory milieu. Present results show that in cocultures with allogeneic and autologous PBMCs, AS/ASCs inhibit the proliferative response of CD4^+^ and CD8^+^ T cells with similar potency (Figures 8(a)–8(f)). Moreover, in both allogeneic and autologous cocultures, similar concentrations of PGE_2_ were found (Figure 8(h)), and kynurenine generation was even higher in autologous than allogeneic conditions (Figure 8(g)).

Our study has some limitations. Due to the gating strategy, we have used, the small contamination in the CD4+ or CD8+ subsets by other populations cannot be excluded.

## 5. Conclusions

In summary, we report, for the first time, that mesenchymal stem cells derived from adipose tissue of ankylosing spondylitis patients (AS/ASCs) are effective suppressors of both allogeneic and autologous CD4^+^ and CD8^+^ T cells, and, similarly to their counterparts from healthy donors (HD/ASCs), exert this antiproliferative effect mainly via soluble factors (kynurenines and PGE_2_). Moreover, some distinctions between the antiproliferative activity of HD/ASCs and AS/ASCs suggest in vivo licensing of the latter cells.

## Figures and Tables

**Table 1:** Demographic and clinical characteristics of the patients.

Parameters	Ankylosing spondylitis (AS) (*n* = 20)
Demographics	
Age, years	42.0 (25-70)
Sex, female (F)/male (M), *n*	8F/12M
Disease duration, years	6 (1.5-18)
Clinical data	
BASDAI, score	6.1 (1.0–8.2)
ASDAS_CRP_, score	3.8 (1–4.7)
BASFI, score	5.0 (0–9.6)
BASMI, score	4.2 (0.25–7.2)
HAQ, score	1.125 (0–2.75)
Laboratory values	
CRP, mg/l	7 (5–59)
ESR, mm/h	15 (1–59)
Medications, %	
NSAIDs	80.0
Nonbiologic DMARDs	30.0
Glucocorticosteroids	10.0

## Data Availability

The data used to support the findings of this study are included within the article.

## Supplementary Materials

Supplementary Materials Figure 1S: exemplary histograms showing PHA-triggered proliferation of peripheral blood mononuclear cells (PBMCs) in various culture variants: (A) proliferation of CD4+ cells; (b) the proliferation of CD8+ cells. ASCs: adipose-derived mesenchymal stem cells; TI: TNF + IFN*γ* cell treatment. Figure 2S: the degree of T cell proliferation inhibition depends on the PBMCs donor. Two HD/ASCs lines (A, C) and two AS/ASCs lines obtained from 2 AS patients (B, D) were cocultured with PBMCs of four different donors (donors 1-4). Coculture condition as in Figure 1. Figure 3S: secretion of IL-10 in PHA-stimulated peripheral blood mononuclear cells cultured separately (PBMCs) or cocultured with ASCs of healthy donors (HD/ASCs) and AS patients (AS/ASCs). Cell preparation and culture conditions as described in Material and Methods. Untreated and TNF + IFN*γ*- (TI-) treated ASCs were used. There were no significant differences between the control of separate cultures of PBMCs and cell cocultures. #*P* = 0.05‐0.01 for intergroup (HD vs. AS) comparison. Figure 4S: correlation of kynurenines and PGE_2_ concentrations with the number of proliferating T cells. Cell preparation, culture conditions, evaluation of CD4^+^ (A, C, E, G) and CD8^+^ (B, D, F, H) cell proliferation, and the measurement of kynurenines and PGE_2_ concentrations as in Figure 5, except that only untreated ASCs were used. Spearman's rank (Rs) (A, B, E, F) and Pearson's (*R*) (C, D, G, H) correlation coefficients and *P* values are shown. Other explanations as in Figure 1. Figure 5S: lack of inverse correlation of IL-10 concentrations with the number of proliferating T cells. Cell preparation, culture conditions, and evaluation of CD4^+^ (A, C, E, G) and CD8^+^ (B, D, F, H) cell proliferation in the presence of untreated and TI-treated ASCs as in Figures 5 and 4S, respectively. The measurement of IL-10 concentrations in culture supernatants was performed as described in Material and Methods. Pearson's (*R*) correlation coefficients and *P* values are shown. Other explanations as in Figure 1.
