# Supplementary figures and images for: Inhibition of Allogeneic and Autologous T Cell Proliferation by Adipose-Derived Mesenchymal Stem Cells of Ankylosing Spondylitis Patients
Source: Stem Cells Int. 2021 Mar 12;2021:6637328. doi: 10.1155/2021/6637328 (PMC7979299; doi:10.1155/2021/6637328)

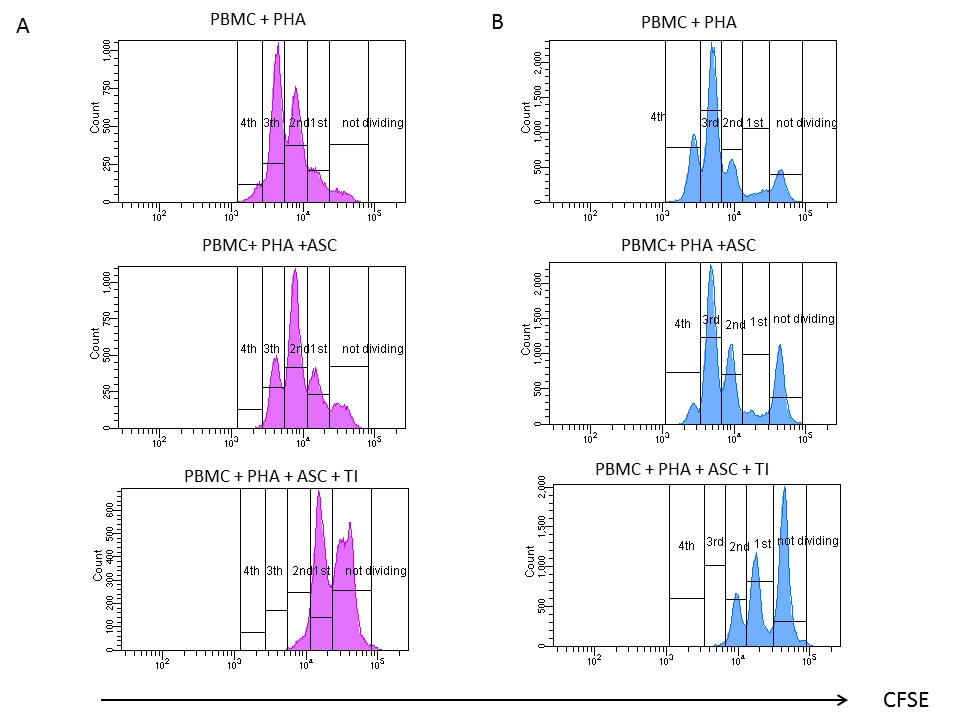


Supp Figure 1S


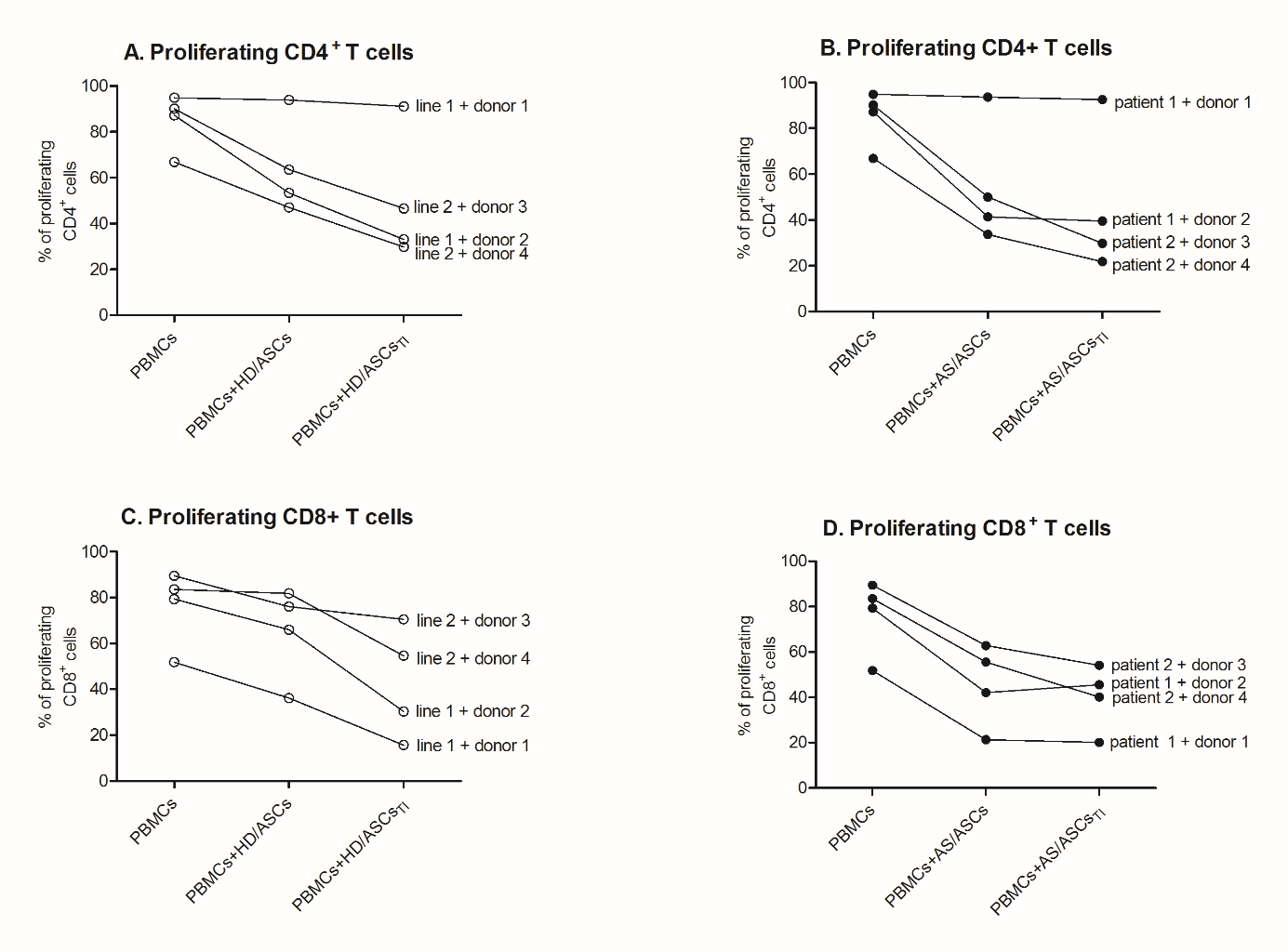


Supp Figure 2S


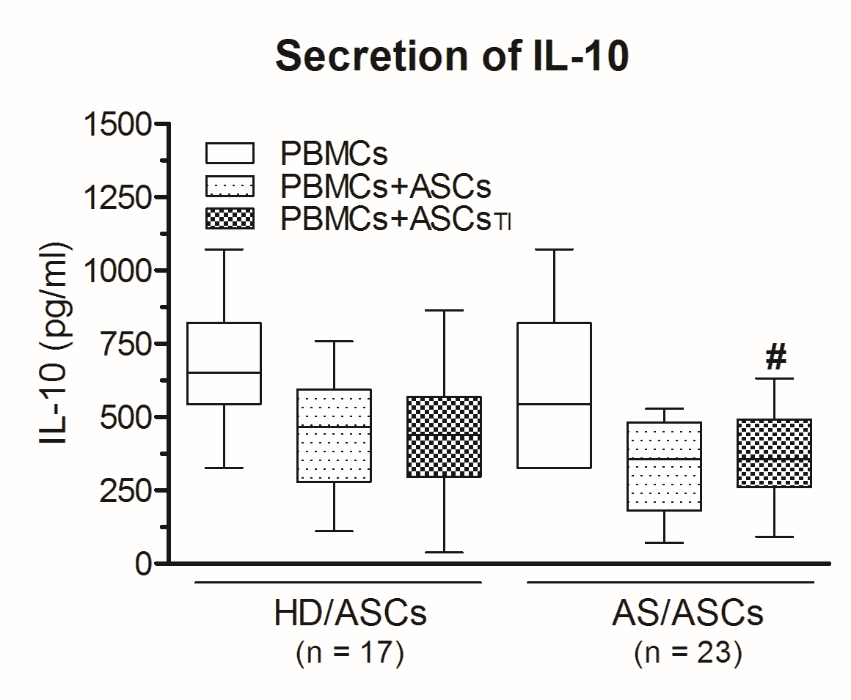


Supp Figure 3S


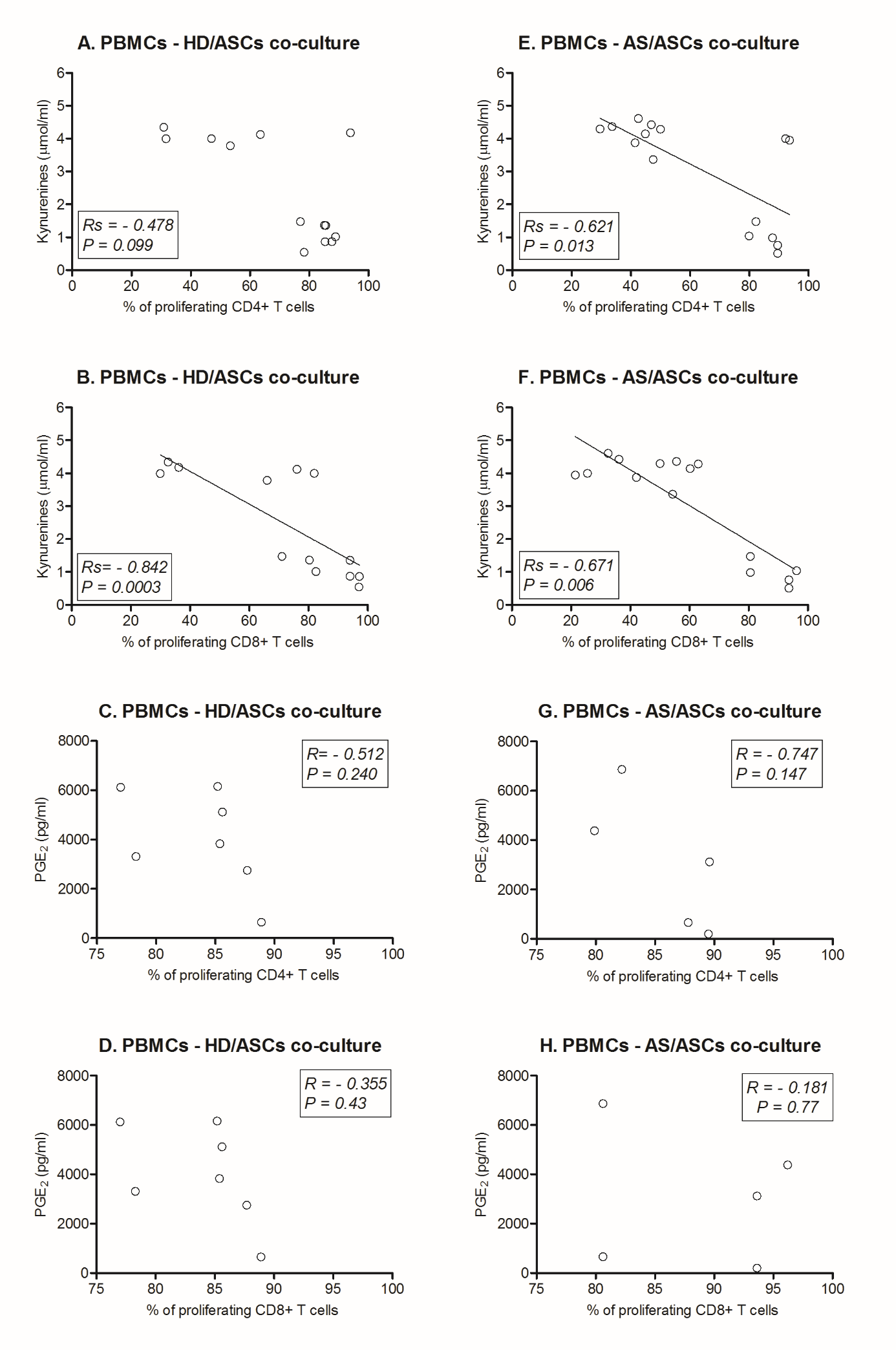


Supp Figure 4S


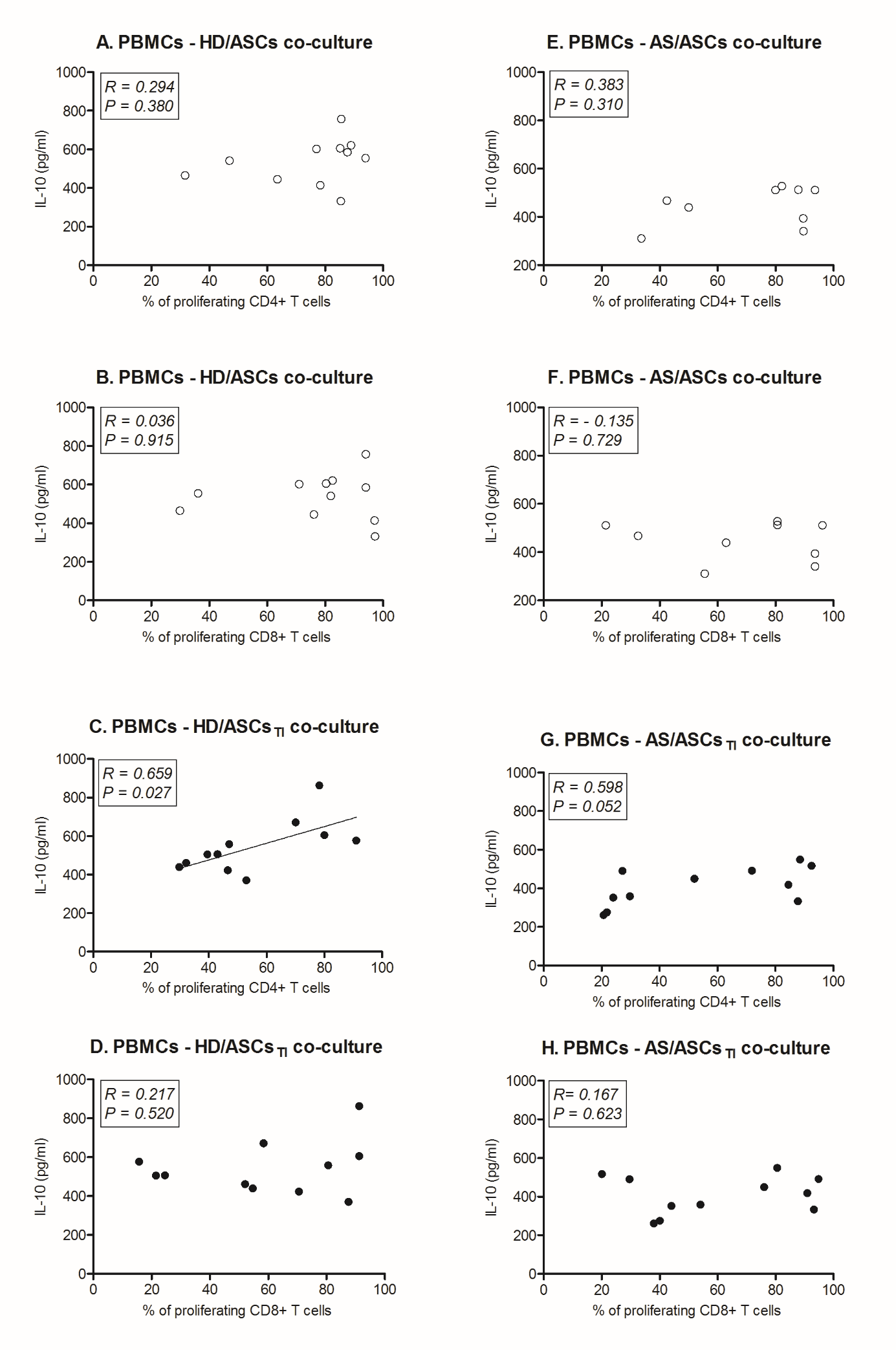


Supp Figure 5S

Supplement: Supplementary Materials — Figure 1S: exemplary histograms showing PHA-triggered proliferation of peripheral blood mononuclear cells (PBMCs) in various culture variants: (A) proliferation of CD4+ cells; (b) the proliferation of CD8+ cells. ASCs: adipose-derived mesenchymal stem cells; TI: TNF + IFNγ cell treatment. Figure 2S: the degree of T cell proliferation inhibition depends on the PBMCs donor. Two HD/ASCs lines (A, C) and two AS/ASCs lines obtained from 2 AS patients (B, D) were cocultured with PBMCs of four different donors (donors 1-4). Coculture condition as in Figure 1. Figure 3S: secretion of IL-10 in PHA-stimulated peripheral blood mononuclear cells cultured separately (PBMCs) or cocultured with ASCs of healthy donors (HD/ASCs) and AS patients (AS/ASCs). Cell preparation and culture conditions as described in Material and Methods. Untreated and TNF + IFNγ- (TI-) treated ASCs were used. There were no significant differences between the control of separate cultures of PBMCs and cell cocultures. #P = 0.05‐0.01 for intergroup (HD vs. AS) comparison. Figure 4S: correlation of kynurenines and PGE2 concentrations with the number of proliferating T cells. Cell preparation, culture conditions, evaluation of CD4+ (A, C, E, G) and CD8+ (B, D, F, H) cell proliferation, and the measurement of kynurenines and PGE2 concentrations as in Figure 5, except that only untreated ASCs were used. Spearman's rank (Rs) (A, B, E, F) and Pearson's (R) (C, D, G, H) correlation coefficients and P values are shown. Other explanations as in Figure 1. Figure 5S: lack of inverse correlation of IL-10 concentrations with the number of proliferating T cells. Cell preparation, culture conditions, and evaluation of CD4+ (A, C, E, G) and CD8+ (B, D, F, H) cell proliferation in the presence of untreated and TI-treated ASCs as in Figures 5 and 4S, respectively. The measurement of IL-10 concentrations in culture supernatants was performed as described in Material and Methods. Pearson's (R) correlation coefficients and P val [file 6637328.f1.docx]
